# The Role of General Practitioners in Actinic Keratosis Management in Europe: A Dermato-Oncologists Initiative

**DOI:** 10.3390/jcm15166467

**Published:** 2026-08-21

**Authors:** Claas Ulrich, Markus V. Heppt, Giuseppe Argenziano, Maria Concetta Fargnoli, Carla Ferrándiz-Pulido, Thomas Jouary, Aimilios Lallas, César Martins, Klara Mosterd, Philippe Saiag, Nathalie Bégeault, Alba Crespo-Cruz

**Affiliations:** 1 Collegium Medicum GmbH, 10117 Berlin, Germany; 2 Charité—Universitätsmedizin Berlin, Institut für Allergieforschung IFA & Fraunhofer Standort Immunologie und Allergologie IA, 10117 Berlin, Germany; 3 Department of Dermatology, Deutsches Zentrum Immuntherapie (DZI), Uniklinikum Erlangen, Friedrich-Alexander-Universität Erlangen-Nürnberg (FAU), 91054 Erlangen, Germany; markus.heppt@uk-erlangen.de; 4 Comprehensive Cancer Center, Erlangen-European Metropolitan Area of Nuremberg (CC ER-EMN), 91054 Erlangen, Germany; 5 Bavarian Cancer Research Center (BZKF), 91054 Erlangen, Germany; 6 Dermpath München, Laboratory for Dermatopathology, Oral Pathology and Molecular Pathology, 80335 Munich, Germany; 7 Dermatology Unit, University of Campania, 80138 Naples, Italy; 8 San Gallicano Dermatological Institute, IRCCS, 00144 Rome, Italy; 9 Dermatology Department, Hospital Universitari Vall d’Hebron, 08035 Barcelona, Spain; 10 Dermatology Unit, Hospital Francois Mitterrand, 64000 Pau, France; 11 First Department of Dermatology and Venereology, School of Medicine, Faculty of Health Sciences, Aristotle University, 54124 Thessaloniki, Greece; 12 Department of Dermatology, ULS Médio Tejo, 2350 Torres Novas, Portugal; wmed.medicos@sapo.pt; 13 Department of Dermatology, Maastricht University Medical Center+, 6202 AZ Maastricht, The Netherlands; 14 GROW Research Institute for Oncology and Reproduction, Maastricht University, 6200 MD Maastricht, The Netherlands; 15 Department of General and Oncologic Dermatology, Ambroise Paré Hospital, APHP, & EA 4340 “Biomarkers in Cancerology and OncoHematology”, UVSQ, Université Paris-Saclay, 92100 Boulogne-Billancourt, France; 16 Medical Dermatology Department, Pierre Fabre Médicament, Les Cauquillous, 81506 Lavaur, France

**Keywords:** actinic keratosis, general practitioner, teledermatology

## Abstract

**Background/Objectives**: Actinic keratosis (AK) is the key precursor of invasive cutaneous squamous cell carcinoma (cSCC). Rising incidence, ageing populations, and limited dermatologists’ availability, particularly in rural and underserved regions, underscore the need for more inclusive care pathways. As in other common diseases such as diabetes or hypertension, general practitioners (GPs) could play a pivotal role in improving access, early recognition, and management of AK, provided that systemic barriers are addressed. **Methods**: This article synthesises current evidence and expert insights from a European multi-country advisory process, focusing on Italy, France, Germany, Spain, Portugal, the Netherlands, and Greece. The advisory board comprised dermatologists selected based on their clinical expertise and national representativeness, and consensus was reached through structured discussion and iterative review of background materials. **Results/Conclusions**: The study outlines unmet needs, barriers, and enablers for GPs’ involvement and proposes a structured strategy supported by consensus recommendations. It offers a pragmatic model for improving AK care through stronger collaboration between primary care and dermatology. Greater GPs’ involvement may support earlier recognition, better triage, improved access to care, and more efficient use of specialist services, while ensuring that complex or high-risk lesions remain under dermatologists’ oversight. This may help optimise AK care pathways across Europe.

## 1. Introduction

Actinic keratosis (AK) is the most frequent in situ keratinocytic neoplasia in white populations and the most common precursor of invasive cutaneous squamous cell carcinoma (cSCC). Although the malignant transformation risk of any single AK is relatively low—estimated at no more than ~10%—patients typically present with field cancerisation, characterised by multiple disseminated AKs across chronically sun-exposed areas such as the face, scalp, hands, and forearms. In this context, the cumulative risk of progression to invasive cSCC becomes substantially higher, underscoring the need for timely diagnosis and appropriate sustainable management [1,2].

A recent global systematic review and meta-analysis estimated the overall point prevalence of AK at 14% (95% CI 14–15), rising to 11–26% in temperate regions, with the Netherlands among the highest-prevalence settings and more than one billion people affected worldwide [3]. Country-level data, although methodologically heterogeneous, illustrate this gradient: in the Netherlands, the population-based Rotterdam Study reported an AK prevalence of 49% in men and 28% in women aged ≥45 years [4]; in Germany, the standardised prevalence was 2.7% overall but reached 11.5% at 60–70 years [5]; and in Spain, 28.6% of dermatology outpatients aged ≥45 years had AK [6]. Comparable population-level data remain scarce for several of the countries represented here, notably Greece and Portugal, underscoring the need for harmonised European epidemiological surveillance. With an estimated incidence of approximately 1928 per 100,000 person-years [3] and continued population ageing, the burden of AK is projected to rise further.

Despite this high prevalence, only approximately half of patients with AK are currently managed according to clinical guidelines, highlighting a substantial treatment gap [1,2]. While according to current standards, dermatologists remain central to AK diagnosis and treatment, the limited availability of specialists and long waiting times create barriers to timely patient care. In several countries, particularly in rural or socially impaired areas, dermatological care deserts exacerbate these issues. This, coupled with often long waiting times for dermatology appointments and a documented shortage or uneven distribution of dermatologists, necessitates exploring alternative management pathways [7,8]. General practitioners (GPs), as the first point of medical contact, could relieve this bottleneck by managing straightforward patients with AK, counselling on treatment adherence, and providing follow-up [7]. Involving GPs in diagnosing and managing in the AK patient journey emerges as a potential solution, though it presents considerable challenges and varies significantly across European nations. Despite the clear need, GPs’ involvement remains inconsistent across European countries. Some healthcare systems (e.g., Spain, Portugal, the Netherlands) support broader GP participation through reimbursement and training, while others (e.g., Italy, Germany, France, Greece) impose regulatory or structural restrictions. Understanding these differences and identifying strategies for scalable GPs integration is central to reducing disparities in care [7,8].

## 2. Materials and Methods

To address the variability in primary care structures and dermatological referral pathways across Europe, an advisory board was convened to collect expert opinions and reach consensus on strategies to improve the management of AKs and to define the potential role of GPs in AK care across Europe. The board included one dermatologist each from Spain, Portugal, the Netherlands, and Greece, and two dermatologists each from Germany, France and Italy, each representing the specific clinical practices, healthcare structures, and challenges of their country, ensuring that the discussion reflected as much as possible the diversity of European healthcare systems.

Experts were selected based on their recognised clinical expertise in dermatology and dermato-oncology, their active involvement in national and international dermatological and oncodermatological societies, and their familiarity with the primary care interface in their respective countries.

**Figure 1:**
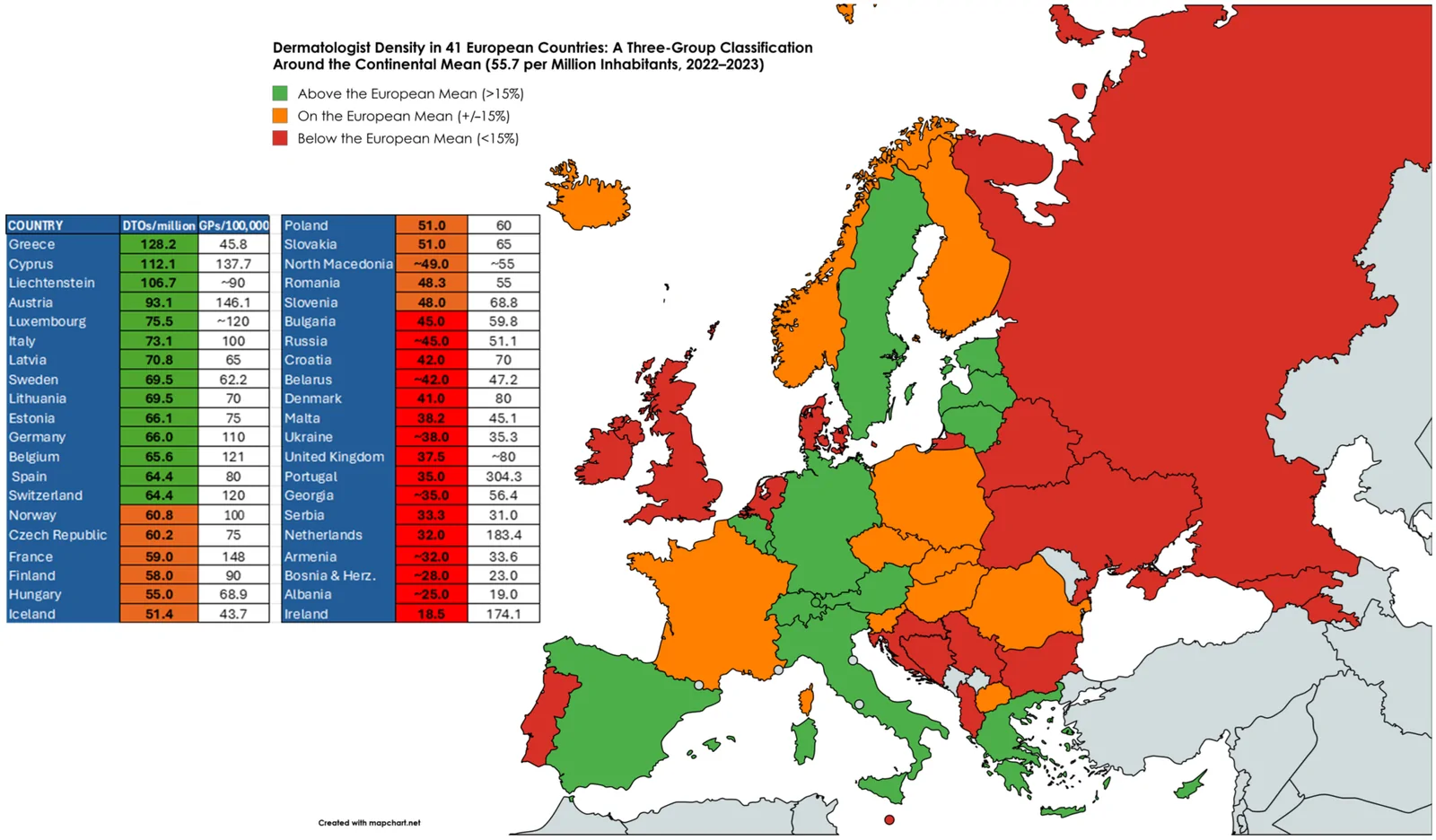
Dermatologist and general practitioner density per 100,000 inhabitants in 41 European countries: three-tier classification relative to the unweighted arithmetic mean (2022–2023). Countries are classified into three groups based on dermatologist density relative to the unweighted arithmetic mean calculated across 41 European countries (5.57 per 100,000 inhabitants): above mean (>15% above the mean: >6.41/100,000), around mean (within ±15%: 4.73–6.41/100,000), and below mean (<15% below the mean: <4.73/100,000). General practitioner (GP) density per 100,000 inhabitants is reported alongside dermatologist density for each country. The ratio of GPs per dermatologist is provided as a relative shortage indicator; higher values reflect higher scarcity of dermatologists relative to the primary care workforce. The areas shown in grey are either territories outside the European continent or, in the case of European or transcontinental territories, those for which we have no data. Dermatologist density data were obtained from EuropeInNumbers.com, which aggregates data from the World Health Organization (WHO) and Eurostat. GP density data were sourced from the Eurostat dataset hlth_rs_physreg, the WHO European Health Information Gateway (Health for All Database), and the World Population Review [9,10,11,12,13]. The European map was created with mapchart.net.

The advisory board met in one structured session, during which background materials were reviewed through a structured 15 min presentation, delivered to the expert panel, summarising the current state of the literature on the role of GPs in the management of AK within European healthcare systems. The review drew upon peer-reviewed publications and complementary epidemiological data and covered the following domains: (i) variability in GP participation in dermatological care across European countries, including country-specific findings; (ii) availability and use of diagnostic tools, including dermoscopy and teledermatology; (iii) national reimbursement frameworks and regulatory contexts; and (iv) existing models of GP-led dermatological care, including international examples. The presentation was designed to establish a shared evidence base among participants and to frame the subsequent consensus-building discussions. To ensure the accuracy and relevance of the data presented, the most recently available data banks were systematically reviewed, and the corresponding figures have been subsequently updated (Figure 1).

Upon completion of the presentation, all panel members contributed to the ensuing discussion in a balanced manner, offering country-specific observations and contextual interpretations of the reviewed evidence. Recommendations were iteratively discussed until consensus was reached. Consensus was defined as agreement by a majority of participants, with dissenting views documented where applicable. Recommendations supported by the published evidence are explicitly distinguished from those based primarily on expert opinion throughout this manuscript.

## 3. Results

### 3.1. Pan-European Distribution of Dermatologists and General Practitioners

This analysis revealed substantial heterogeneity across Europe. Countries with the highest dermatologist density including Greece (12.82/100,000), Cyprus (11.21/100,000), and Austria (9.31/100,000) tend to operate specialist-centred healthcare systems with limited GP gatekeeping. Conversely, countries with the lowest dermatologist density, including Ireland (1.85/100,000), the Netherlands (3.20/100,000), and the United Kingdom (3.75/100,000) maintain strong primary care gatekeeping models with high GP density. Among the seven countries represented in the advisory board, the dermatologist density ranged from 3.20/100,000 (Netherlands) to 12.82/100,000 (Greece), reflecting differences in healthcare organisation, training opportunities, and referral pathways and illustrating the diversity of healthcare contexts within which GP involvement in AK management must be considered. These disparities underscore that strategies to expand GP involvement in AK care must be adapted to national healthcare structures rather than applied uniformly across Europe. It is worth acknowledging that dermatologist density data for Liechtenstein, Luxembourg, Finland, Poland, North Macedonia, Russia, Belarus, Ukraine, Georgia, Armenia, Bosnia and Herzegovina, Albania, and the United Kingdom were not directly available from consolidated international databases. These figures were estimated based on total physician density ratios and regional benchmarks derived from WHO [11] and World Bank Health Data [13] and should be interpreted as approximations. The mean dermatologist density reported (5.57 per 100,000 inhabitants) represents an unweighted arithmetic mean across the 41 countries included in this analysis and does not constitute an officially published European statistic.

General-practitioner availability shows an equally marked and partly inverse gradient. According to Eurostat 2023 data, the density of practising generalist medical practitioners ranged from among the highest in the Netherlands (183.4 per 100,000 inhabitants) to among the lowest in Greece (45.8 per 100,000), the latter consistent with the strongly specialist-centred model observed in Greece. These figures require cautious interpretation, as some countries report generalists licensed to practise rather than those actively practising; Portugal, for example, records a high nominal figure (304.3 per 100,000, licensed) that masks a well-documented shortage and uneven geographic distribution of practising GPs. This heterogeneity is central to the feasibility of GP-integrated models: where GPs are themselves scarce or maldistributed, as in Portugal, the proposed shared-care approach faces significant operational constraints [14].

### 3.2. Overview of the Literature Review Presented to Advisory Board Participants

The preliminary literature review presented to participants highlighted substantial variability across European countries in the feasibility and extent of GP participation in dermatological care [9,10,11,12,13]. Evidence from multiple European settings indicates that GPs can safely and effectively contribute to AK diagnosis, triage, and treatment when provided with appropriate training, reimbursement structures, and access to specialist support [15,16,17,18]. Nations such as the Netherlands, Spain, Portugal, and the United Kingdom have developed more integrated frameworks enabling primary care participation in dermatological management, whereas countries including Italy and Germany face persistent cultural, regulatory, and infrastructural barriers [15,16]. EADV reports have identified the integration of GPs into dermatological care—particularly in rural and underserved regions—as a priority, emphasising the potential of dermoscopy training, teledermatology, and structured treatment guidelines to support this transition [19]. The use of teledermatology was highlighted as both a short-term solution for access gaps and a long-term strategy for integrated care delivery [20,21,22,23]. Reimbursement policies were identified in the literature as a critical enabling or limiting factor for GP involvement in AK management across European healthcare systems [17,18]. These findings framed the subsequent advisory board discussions and informed the development of strategic pillars and action plans, as detailed below.

### 3.3. Country-Specific Findings from the Advisory Board

#### 3.3.1. Italy

Italy represents one of the most restrictive settings regarding GP involvement in AK management. Regulatory barriers, particularly Nota 95, limit the prescription of topical therapies for AK exclusively to dermatologists, effectively excluding GPs from active treatment. Additionally, limited access to dermoscopy and cryotherapy further constrains their already restricted role in AK care. While many GPs are motivated, meaningful involvement will require systemic regulatory and structural reform.

#### 3.3.2. Greece

In Greece, the relatively high number of dermatologists compared to the European average (130 vs. 56 dermatologists per million of inhabitants) allows direct access to specialised care. Thus, AK management is almost exclusively led by dermatologists. GPs receive much less training and have limited access to diagnostic tools and reimbursement, which restrict their role. As a result, dermatologists manage the overwhelming majority of AK cases, reflecting a strongly specialist-centred healthcare model.

#### 3.3.3. Germany

In Germany, there is some scepticism from dermatologists about GP involvement. Access to dermoscopy and cryotherapy is limited in primary care, and teledermatology often bypasses GPs. Nonetheless, GPs express motivation for training, and simplified topical treatment protocols could offer a starting point for collaboration.

#### 3.3.4. France

France faces long waiting times and regional gaps in dermatologists’ distribution. Teledermatology is reimbursed, but GPs’ involvement remains limited due to a lack of equipment and confidence in side-effect management. But the younger generation of GPs seems to show growing interest, and national congresses have included GP-focused sessions, suggesting readiness for targeted interventions. Examples include structured training programs, standardised clinical algorithms for AK diagnosis and management, improved access to dermatoscopy and treatment tools, and strengthened collaboration pathways with dermatologists through tele-expertise models.

#### 3.3.5. Spain

Spain demonstrates robust GP participation, although with marked regional variability. In several areas, communication between GPs and dermatologists is well established through structured teledermatology programs, facilitating efficient triage and shared management. In some regions, GPs routinely prescribe topical treatments for AK and use dermoscopy and cryotherapy in daily practice. Continuing medical education initiatives jointly organised by dermatologists and particularly engaged GPs further strengthen this collaboration, enhancing communication, confidence, and clinical empowerment. However, in other regions of the Spanish territory, implementation remains more limited, and GPs may be more reluctant to adopt these tools. The key challenge lies in achieving greater standardisation and ensuring consistent adherence to guidelines across regions.

#### 3.3.6. Portugal

The figures retrieved from the consulted databases corroborate the expert-based assessments, reinforcing the validity of the observed trends: insufficient GP availability and their uneven distribution across regions critically hinder the widespread adoption and sustained delivery of care. On a positive note, Portugal allows GPs to prescribe commonly used AK treatments within standard reimbursement pathways, and GPs can manage many straightforward cases in primary care, particularly when diagnosis is clinically typical. Furthermore, teledermatology-supported referral pathways within Portugal’s National Health Service, combined with structured mentorship and training links with dermatology, could strengthen GP confidence, improve triage accuracy, and enable timely and safe escalation of complex or uncertain lesions.

#### 3.3.7. The Netherlands

In the Netherlands, a structured guideline for the management of low-risk skin cancer and precursors is available for GPs. However, only a small proportion of AK cases are GP managed, suggesting low confidence of GPs in their own knowledge and time constraints. There are shared-care models, such as the 1.5-line care, in which GPs manage straightforward cases with active support and validation from dermatologists via teledermatology or structured mentorship. This shared-care framework aims to balance the accessibility of primary care with specialist clinical oversight, enhancing GPs’ confidence and diagnostic accuracy while preserving quality of care.

### 3.4. Strategic Actions to Involve GPs

The experts proposed a staged strategy for GP integration based on five pillars (Table 1). Highlighting the importance of starting with motivated GPs and expanding based on demonstrated success, advisory board members recommend the following: (1) properly educating and training GPs, (2) engaging GPs through direct and indirect benefits for them and their patients, (3) creating a network to support expertise and referral, (4) providing GPs with simple practical guidelines for AK management, and (5) developing patient education initiatives.

### 3.5. A Practical Care Pathway for GP-Led AK Management

To complement the strategic framework summarised in Table 1, the advisory board distilled a concise practical pathway to support GPs in day-to-day AK care, delineating the diagnostic, therapeutic, and referral steps that can be safely undertaken in primary care. These recommendations are consistent with current European and national guidelines [24,25].

**Diagnosis and biopsy.** AK is primarily a clinical and dermoscopic diagnosis, and typical flat scaly lesions on chronically sun-exposed skin do not require histological confirmation in primary care. A biopsy preferably of the thickest, indurated, tender, rapidly growing, bleeding, or ulcerated lesion should be performed whenever invasive cSCC is suspected, when the clinical diagnosis is uncertain, when a lesion has not previously been characterised, or when a lesion fails to respond to appropriate field-directed therapy.

**Treatments suitable for primary care.** For a limited number of discrete (grade I–II) lesions, lesion-directed cryotherapy is appropriate and widely available. For multiple lesions or field cancerisation, field-directed topical therapies (e.g., 5-fluorouracil, imiquimod, tirbanibulin, or diclofenac) or daylight photodynamic therapy are preferred, with the choice guided by the lesion number and distribution, patient adherence, and tolerability. GPs should be equipped to counsel patients on the expected local skin reactions and to manage common side effects, as this is central to treatment adherence and success.

**When to refer.** Referral to dermatology is recommended for extensive or treatment-refractory field cancerisation; immunosuppressed patients, including solid-organ transplant recipients; any suspicion of invasive cSCC or other skin malignancy; diagnostic uncertainty; lesions at high-risk or functionally and cosmetically sensitive sites (e.g., lip, ear, or periocular region); and lesions that recur or fail to respond to first-line therapy. Teledermatology-supported triage can facilitate timely appropriate escalation while avoiding unnecessary referrals.

## 4. Discussion

In light of the rising skin cancer incidence, leading dermatological experts are emphasising the importance of the early detection of skin tumours through skin cancer screening campaigns. While this strategy has proven successful, at least regionally, in the early detection of malignant melanoma, only half of patients with actinic keratosis are currently treated according to the guidelines [26]. Current statistics also show that the increase in invasive cSCC in countries with predominantly white populations is significantly higher than the increase in new cases of melanoma. Since the number of patients with AK far exceeds the therapeutic capacity of dermatology departments in most European countries, the active involvement of GPs appears essential, not only in general skin cancer screening but also in the management of carcinoma in situ lesions such as actinic keratosis.

Across Europe, the literature and expert discussions suggest that GPs could play a stronger role in diagnosis, triage, and treatment, particularly for AK, if appropriate training, reimbursement, and support structures are provided. While countries such as the Netherlands and the United Kingdom have integrated GPs more substantially into primary dermatologic care, other nations remain reliant on specialist dermatologists [15,16]. Studies have also shown that trained GPs can safely and effectively manage AK using basic diagnostic tools and first-line treatments, provided they have access to specialist support when needed [17,18]. A recurring point of debate in the literature and in contemporary consensus-building efforts is whether GPs are adequately trained, properly equipped, and sufficiently supported within their healthcare systems to assume a more independent role in the management of AKs [20,21]. Based on these statements and considering a country’s specific healthcare structure and regulatory context, the experts collectively identified priorities and practical next steps to improve GPs’ involvement in AK management. The objective was to establish a shared framework outlining common goals and actionable priorities that can be adapted and implemented nationally. The advisory board process revealed several overarching themes that cut across national contexts. There was unanimous agreement that the current dermatologist workforce is insufficient to meet the growing demand and necessity for AK care, particularly in rural and underserved regions.

While the fear of misdiagnosis or under-treatment exists, it is often counterbalanced by studies indicating that properly trained GPs can effectively identify and treat AK using cryotherapy or topical agents. The use of dermoscopy and teledermatology also enhances diagnostic accuracy, enabling efficient triage to dermatologists when necessary [20,21,22,23]. Additionally, all countries acknowledged the importance of training and equipping GPs with basic dermatological skills, such as the clinical recognition of AK and side-effect management for topical AK therapies. However, access to dermoscopy and modern AK treatment approaches remains inconsistent, with some nations (e.g., Spain and Portugal) reporting more established practices, while others (Italy, Germany, Greece) remain limited by structural or regulatory barriers. The five strategic pillars proposed in Table 1 are broadly consistent with the evidence from the published literature. The emphasis on GP education and training aligns with findings from Noels et al., who demonstrated that GPs with targeted dermatological training showed improved confidence and diagnostic accuracy in AK management [22,23]. Evidence from primary-care dermatology studies suggests that such integrated models can improve early detection and treatment uptake, if GPs receive ongoing targeted training, access to dermatoscopy, and regular expert feedback [23].

Complementary data from statistics further support this variability, showing that the feasibility and extent of GPs’ participation in dermatology vary markedly across European health systems [9,10,11,12,13]. Countries with reimbursement frameworks (France, Spain, Portugal, the Netherlands) demonstrated more mature GPs engagement, while those lacking such structures (Italy, Germany) showed fragmented adoption. Finally, reimbursement policies emerged as the single most important enabler or barrier. Where GPs were authorised and reimbursed for prescribing AK treatments (Portugal, the Netherlands), their involvement was significant. On the other hand, the commitment of German GPs to the management of AKs is still in need of improvement, despite analogous reimbursement options. Conversely, restrictive reimbursement and prescribing rules in Italy effectively exclude GPs from the AK care pathway, despite demonstrated willingness and motivation.

Data coming from the DIADERM study dataset, a nationally representative Spanish registry, further quantify the opportunity to streamline care. The study, which analysed more than 15,000 first-time dermatology visits across multiple regions, found that approximately 31.6% of referrals from primary care were classified as potentially avoidable. These cases most involved benign or low-risk conditions such as seborrheic keratoses, melanocytic nevi, and actinic keratoses—lesions that, with appropriate training and clinical support, could often be assessed and managed directly in primary care. Importantly, patients in the “potentially avoidable referral” group were discharged at the first dermatology visit in 69.7% of cases, compared with only 39.1% in necessary referrals, demonstrating that specialists were dedicating a large proportion of clinic time to conditions requiring no further intervention [27]. This pattern reflects a structural misallocation of resources: dermatologists are overwhelmed with benign cases, while patients with high-risk oncologic lesions may face longer waiting times. The inclusion of AK as one of the most frequent avoidable referrals diagnoses further underscores the opportunity for primary-care management, particularly given the availability of straightforward effective topical therapies and the suitability of AK for teledermatology supported triage.

The recommendation to establish teledermatology-supported networks reflects evidence from Ferrándiz et al., who showed that teledermatology-driven prescribing of topical AK therapies achieved clinical outcomes comparable to face-to-face management [26]. The evidence strongly supports the integration of teledermatology as a safe and effective adjunct for AK care. Ferrándiz et al. demonstrated that when imiquimod was prescribed through a teledermatology pathway, the clinical outcomes were comparable to face-to-face management [26]. Teledermatology was consistently recognised as a pivotal tool for GP–specialist collaboration. The experts highlighted teledermatology as both a short-term solution for access gaps and a long-term strategy for integrated dermatology care. Development of hybrid teledermatology, mentorship, and collaborative care frameworks are concrete examples of potential supporting measures to improve GP involvement. However, while teledermatology can maintain diagnostic and therapeutic quality, it should not function as a standalone modality. Instead, optimal outcomes are achieved through blended shared-care models, in which GPs provide local counselling, adherence support, and side-effect monitoring, while dermatologists offer remote oversight. Such integrated pathways ensure both the effectiveness of AK therapy and the real-world support needed for successful treatment [26]. The evidence and expert opinion also highlight that teledermatology is not limited to rural contexts. In urban settings with high demand and long queues, structured tele-expertise embedded in primary care can improve the equity of access and reduce waiting times, provided workflows, reimbursement, and image quality standards are in place. These insights support European strategies that integrate teledermatology into GP-led pathways while preserving rapid escalation for atypical or high-risk lesions [28]. Furthermore, integrating tele-expertise—where dermatologists remotely validate GP assessments—would enable safe task-shifting without compromising the diagnostic accuracy. In this context, the example of the DIADERM study in Spain demonstrates not only the clinical feasibility but also the system-level value of empowering GPs in AK management [27]. The referral criteria and shared-care model proposed by the experts are consistent with the 1.5-line care framework successfully implemented in the Netherlands [23]. Despite its promise, teledermatology also presents important challenges that must be addressed for successful implementation. These include (1) technical limitations, such as the variability in image quality and the absence of standardised capture protocols; (2) reimbursement barriers, as many European countries lack clear billing frameworks for asynchronous tele-expertise consultations; (3) infrastructure requirements, including reliable internet connectivity and compatible devices, which remain unevenly distributed across rural and urban settings; (4) diagnostic reliability concerns, particularly for lesions requiring tactile assessment or where image resolution is insufficient; and (5) medicolegal considerations, including questions of liability when a diagnosis or treatment decision is made remotely without direct patient examination. Addressing these challenges through national regulatory frameworks, standardised protocols, and appropriate reimbursement structures will be essential to realising the full potential of teledermatology in GP-led AK care [29,30,31,32,33]. However, it should be noted that several recommendations—particularly those relating to reimbursement reform and regulatory change—are based primarily on expert consensus rather than the published evidence, and their implementation will require national-level policy engagement.

European initiatives such as European Academy of Dermatology and Venereology (EADV) reports have suggested that the integration of GPs into dermatological care should be a priority. Additionally, the EADV has highlighted gaps in AK treatment and the need for a multidisciplinary approach, particularly in rural and underserved regions [19]. The scarcity of dermatologists in such areas combined with long waiting times in urban centres justify a system that empowers GPs through dermoscopy training, teledermatology integration, and structured treatment guidelines. As an example and potential model, the success of GP-led interventions in New Zealand illustrates the effectiveness of this approach [19].

While existing consensus-based interdisciplinary guidelines for AK management [24] provide a robust clinical foundation, their complexity and specialist-oriented framing may limit their direct applicability in primary care settings. Adapting these guidelines into concise practical protocols, focused on topical treatments and tailored to the diagnostic and therapeutic scope of GPs, is therefore a prerequisite recommended by the experts for enabling safe and confident GP-led AK management (Table 1).

Measures like structured education, teledermatology and reimbursement alignment could expand access and improve outcomes by empowering GPs to participate in AK management. While health system studies underscore its broader value in enhancing equity of access, one of the most obvious measures concerns teledermatology. As the equipment and frameworks already exist in many countries and could be easily expanded, embedding teledermatology within GP practices, supported by reimbursement and standardised guidelines could offer a pragmatic path to expand the capacity. Collectively, these findings provide strong evidence for a model where GPs manage routine AK, supported by teledermatology and specialist oversight. This aligns with European policy goals of strengthening primary care while achieving efficiency and maintaining high quality and safety in dermatology care [28].

The dermatologist workforce gap creates opportunities for GPs to contribute more substantially to diagnosis, treatment, and patient education. Patient education represents an essential and often underutilised component of effective AK management, directly impacting treatment compliance and long-term outcomes. The advisory board identified the need to develop targeted campaigns for high-risk AK populations, with a focus on educating patients on AK as a precancerous condition, treatment expectations, and sun protection behaviours. GPs are uniquely positioned to deliver such education at the point of care, given their longitudinal relationships with patients and their central role in preventive medicine. This is particularly critical given that only half of patients with AK are currently treated according to the guidelines [26], suggesting significant gaps in patient awareness and engagement. Raising awareness through media, community talks, and patient-facing information materials—including the use of digital channels and community influencers—has been proposed as a pragmatic approach to reach high-risk populations at scale [19]. Such initiatives are consistent with EADV recommendations emphasising the need for a multidisciplinary approach to AK management, in which patient empowerment and education are integral components of the care pathway [19]. Embedding structured patient education within GP-led AK care pathways, supported by standardised educational materials, would complement the clinical management strategies outlined in the preceding pillars and contribute to improved treatment adherence and long-term prevention outcomes [17,18].

It should be acknowledged that this initiative did not include expert perspectives from Nordic countries such as Finland and Sweden, where GP-led AK management is already well established. This geographic gap represents a limitation of the current work, as the Nordic experience could offer particularly instructive lessons for countries still transitioning toward primary-care-integrated models. Incorporating these perspectives in future advisory processes would further strengthen the evidence base and cross-national applicability of the proposed framework. Similarly, the dual shortage of both GPs and dermatologists in some countries underlines that no single model is universally applicable, and this must be explicitly acknowledged when designing scalable GP-integration strategies.

## 5. Conclusions

This initiative contributes a structured, multi-country, dermatologist-led consensus that translates the recognised gap between the growing burden of AK and limited specialist capacity into a pragmatic nationally adaptable framework for integrating GPs into AK care, articulated around five strategic pillars: GP education and training, GP engagement, expert network and referral, practical guidelines, and patient education. Its principal added value lies in linking country-specific enablers and barriers to a concrete staged action plan (Table 1) and care pathway, underpinned by teledermatology and clear referral criteria that can inform health-system and policy decisions across heterogeneous European settings. However, as the conclusions are based on expert opinion, further discussions and working groups including GPs would be necessary to integrate their perspectives and provide a credible and practical foundation for cross-national recommendations on GP involvement in AK management.

## Figures and Tables

**Table 1:** Strategic pillars and action plan for GPs’ integration in AK management.

Category	Goal	Key Actions
Education and Training	Equip GPs with knowledge and confidence	In-person initial training (diagnosis, dermoscopy basics, treatment selection, side effects management) Online refreshers/modules Accreditation for continuing medical education (CME) to intensify participation
Motivation and Value Proposition	Encourage GP engagement through relevance and efficiency	Reframe AK treatment as a secondary prevention strategy for cSCC Highlight benefits: fewer repeat visits, better patient access, dermatologists freed for complex cases
Networking and Support	Prevent GP isolation; provide backup	Create local dermatologist–GP networks Set up (reimbursed) teledermatology access points (email, phone consults) Establish clear referral protocols
Guidelines and Protocols	Enable standardised practical care	Provide concise evidence-based treatment protocols tailored to GP scope Focus on topical treatments initially Distribute info sheets for patients and nurses
Patient Education	Improve treatment compliance and awareness	Develop campaigns targeting high-risk AK populations Educate on AK as a pre-cancer, treatment expectations, and prevention (sun protection). Use media, community talks, and influencers

## Data Availability

The data that support the findings of this study are available from the corresponding author upon reasonable request.
